# Crisis in arms control: an introduction

**DOI:** 10.1007/s42597-022-00074-8

**Published:** 2022-05-23

**Authors:** Simone Wisotzki, Ulrich Kühn

**Affiliations:** 1 Leibniz-Institut Hessische Stiftung Friedens- und Konfliktforschung (HSFK), Baseler Straße 27–31, 60329 Frankfurt/Main, Germany; 2 Institut für Friedensforschung und Sicherheitspolitik an der Universität Hamburg, Beim Schlump 83, 20144 Hamburg, Germany

## Introduction

The term “crisis” is widely used in relation to arms control. A simple search in the journal “Arms Control Today” generated more than 6000 hits for headlines featuring the word “crisis”—and that was before Russia attacked neighboring Ukraine on February 24, 2022. Already before the Ukraine War, the COVID-19 pandemic had brought many arms control regimes to a halt. Review conferences and meetings of states were cancelled, the United Nations and its Office of Disarmament (UNODA) were temporarily closed. Now, two years into the pandemic, meetings of states are still being held in hybrid formats with the majority of diplomats and civil society participants holding debates in online fora. Other meetings, such as the Review Conference of the Parties to the Treaty on the Non-Proliferation of Nuclear Weapons (NPT), which was scheduled for August 2021 and had been postponed to January 2022, has been rescheduled again for August 2022. The First Meeting of States Parties of the Treaty on the Prohibition of Nuclear Weapons (TPNW) had to be postponed as well and is now scheduled to take place in June, 2022. The cancellation of the conferences has resulted in delayed or stalled implementation of arms control agreements.

An even more negative development is that arms control agreements are being undermined either by external events, such as the repeated use of chemical weapons in Syria, or, in the case of nuclear arms control, by a lack of compliance among NPT nuclear-weapon states with their Article VI disarmament obligations, against the background of ongoing nuclear modernization. The MacArthur Foundation will cease its funding of nuclear arms control research by 2023, stressing that the step-by-step approach toward nuclear disarmament, which has been pursued since the 1970s, has not been at all successful (Wilson 2021). Multilateral disarmament is also facing major difficulties due to the protracted crises of U.S.-Russian nuclear arms control and conventional arms control in Europe. The Intermediate-Range Nuclear Forces (INF) Treaty, which banned all U.S. and Russian land-based missiles and launchers with ranges between 500 and 5500 km was suspended by the United States in August 2019 in response to alleged Russian noncompliance. A year later, Washington also withdrew from the Open Skies Treaty, citing Russian noncompliance as well. United Nations Secretary General Antonio Guterres summarized the different dimensions of the arms control crisis as follows: “I will be blunt. Key components of the international arms control architecture are collapsing”.^1^

The articles for this Special Issue were written before Russia’s war in the Ukraine. It focuses on the different dimensions of crisis in arms control. It looks at the diverse domains of arms control, including bilateral or multilateral nuclear arms control and disarmament, conventional arms control, humanitarian arms control, as well as arms export controls. It also seeks to answer the question of how the different arms control agreements cope with the external and internal challenges putting them under pressure.

While some of the agreements, such as the NPT, were already concluded during the Cold War, others were only negotiated recently, such as the TPNW or the Arms Trade Treaty (ATT). During the Cold War, arms control was seen as an instrument to stabilize military relations between the superpowers, which would also provide communication channels. When the last General Secretary of the Central Committee of the Communist Party of the Soviet Union, Mikhail Gorbachev, announced his policies of “Glasnost” and “Perestroika” and subsequently sought agreement on mutual arms reduction with the United States, arms control entered its “golden age.” Over the course of the following decades, an unprecedented number of cooperative security institutions in the nuclear, conventional, and confidence-building realm were negotiated and concluded. Today, most of these agreements, such as the INF or the Conventional Armed Forces in Europe (CFE) Treaty, are gone. With past agreements collapsing, the future of U.S.-Russian nuclear arms control looks bleak. While throughout the Cold War, the superpowers shared an interest in cooperating for the sake of survival, this core motive gradually lost appeal in the post-Cold War world (Kühn 2020). Today, new challenges, such as the political and military rise of China, put additional pressure on what little remains of the Cold War era arms control architecture.

Instead, deep-rooted mistrust continues to shape relations between the United States and Russia, which also affects the prospects of multilateral arms control. In 2019, Russia tabled a resolution calling for the UN First Committee to be relocated to either Geneva or Vienna in reaction to diplomats from G‑77 Member States being denied visas. The Conference on Disarmament was unable to agree on an agenda for the first time since 2005 (Wisotzki 2020). Moreover, the repeated use of chemical weapons in Syria has still not been sanctioned due to Russia blocking the UN Security Council. While some experts conclude from this that the Chemical Weapons Convention (CWC) is in a serious crisis, others perceive the attacks as dangerous precedents, but argue that the CWC regime is still both relevant and stable (Jakob 2019). While the changes in world order since the end of the Cold War have had considerable impact on arms control and disarmament, the specific regimes, such as humanitarian arms control, are confronted with changes in the type of conflict and new proliferation challenges. Antipersonnel landmines as well as cluster munitions have been used as well, mostly by non-state armed groups. Moreover, new technologies add another layer of challenges to those already faced by existing arms control regimes, at the same time demonstrating the lack of such regimes. Cyber and artificial intelligence are “invisible” capabilities which could challenge nuclear stability. Sophisticated hypersonic glide vehicles can be launched from normal ballistic missiles but “have greater maneuverability upon reentry, so as to take an unpredictable path and evade defenses en route to a target” (Lissner 2021, p. 11). Finally, with an ongoing war raging in Ukraine, chances for a renewed push for multilateral disarmament and arms control seem even more bleak than before the war.

Diplomats and politicians often attribute the term “crisis” to a situation to justify specific action. Researchers are sometimes more hesitant to use such terms. However, academic research on crisis distinguishes between crisis as an object of observation and as an instrument of observation (Bösch et al. 2020a: 5). It was this distinction that triggered our interest in editing this Special Issue. We asked our authors to describe the different facets of crisis and the responses we received were varied and nuanced. The Special Issue aims to examine the crisis of arms control and its effects on the international order, while at the same time not only describing the crisis empirically, but also fundamentally discussing in comparative terms the question of whether the central concepts of arms control and disarmament, most of which were developed during the East-West conflict or in its immediate aftermath, have become obsolete in the face of new security challenges, including terrorism, and technological innovations. To what extent are existing arms control norms able to respond adaptively to these diverse challenges?

## Crisis research—what is at stake?

Crisis has become “an all-pervasive rhetorical metaphor” (Holton 1987, p. 502). Most researchers would probably agree with this sentence. However, there is strong disagreement on questions such as analytical utility, subjectivity, or normativity of crisis (Bösch et al. 2020b). This introduction seeks to sketch some of the contours of the academic debates on crisis, mainly staying within the framework of international theory. In ancient Greek, the term crisis was central to politics and had different meanings, including “decision” in the sense of reaching a tipping point in political debates—it is a “moment of decisive intervention” (Hay 1999, p. 323). While in classical Greek, crisis includes both domains—the “subjective critique” and the “objective crisis” (Koselleck and Richter 2006, p. 359), today, these two dimensions are often treated separately. Academic crisis research stresses the positive tension between the definition of crisis as an *object of observation*, where crisis is socially constructed by certain actors, and as an *instrument of observation* in order to identify the mechanisms, dynamics, and dimensions of crisis (Bösch et al. 2020a: 5). Pragmatists such as Milstein (2015) seek to revive the reflexive elements of crisis in its original meaning, where the dichotomies between fact and values are blurred and the traditional distinction between analytical science and normative philosophy cannot be upheld. Instead, crisis “presupposes our ability to critically observe and take responsibility for our social world. As such, the modern concept of crisis is an essentially *participatory *concept, whose very invocation calls not just for observation and critical judgment but *action*” (Milstein 2015, p. 143).

While such an analytical distinction might be empirically difficult to identify, academic research also focuses on the status of exceptionality of crisis. In order to perceive crisis as “lived experience,” Hay (1999, p. 323–24) proposes an analytical distinction between failure (understood as an accumulation of contradictions) and crisis (the moment of decisive intervention when those contradictions are detected). Many studies draw on the root meaning of the Greek word “crisis,” which signifies the medical decision about life and death, where the right decision could lead to recovery (Koselleck 1982). Graf (2020) also highlights the evolution of the meaning of the term: While in the 1920s, crisis was perceived as a sort of positive change, this form of agency was lost in the 1970s when crisis was understood as a difficult and dangerous situation with a potentially negative outcome.

A crisis forces an immediate reaction and the suspension of routines and procedures—the term “crisis” evokes a state of emergency. To frame a political problem as a crisis generates public attention and pressure to react (Milstein 2015). In this arena of exceptionalism, announcing a crisis signals an existential threat, but actors are empowered to act and might be able to manage the crisis through their decisions and action. Crisis can also have a policy-enabling function—policies might change or new institutions be built in the aftermath of a crisis. Before the Ottawa Convention was negotiated in 1996, its advocates convincingly depicted antipersonnel mines as causing a humanitarian crisis (Wisotzki 2013). The global nuclear nonproliferation and bilateral arms control regimes also came into being due to the world having survived the Cuban Missile Crisis. Therefore, crisis should also be recognized as an opportunity to manage problems, address deficits, and foster innovation. Different IR approaches stress the exceptionality of crisis rhetoric. Instead of perceiving crisis as an event, social constructivists introduce the concept of “crisis-as-claim” (Spector 2019, p. 277). This change of perspective also implies the need to ask different research questions such as how these claims of urgency enhance the power and interests of the claim makers. When events are framed as crisis, political leaders “attribute meaning to ambiguous contingencies” and make deliberate choices, e.g., declare a situation as threatening, demand exceptional rights and powers for responding to the specific situation (Spector 2019, p. 277). For Kalkman (2019, p. 423), such crisis claims work in similar ways to securitization—crisis claims may also grant people emergency powers and enable leaders to deploy the desired resources (Klenk and Nullmeier 2010). By pointing to specific crises such as 9/11, Widmaier (2007, p. 785) also underlines the role and relevance of interpretative leadership, which defines threats and crises in order to guide societal debates on events in a desired direction. To use the language of Alexander Wendt, crises are in effect, what we make of them, and what we make of them determines how we respond (Hay 2013: 23). The transmission of meaning works through discourse and determines the nature of crisis. Poststructuralists also point to the exceptionalism of crisis situations—R.B.J. Walker (2010), for example, characterizes crises as being an atmosphere of often apocalyptic levels. Critical security studies also highlight the discursive production of an “emergency culture” (Nabers 2017, p. 421).

Among securitization theorists there is also a growing consensus that academic analysis should go beyond a focus on speech acts, and should also concentrate on political practices and routines. Discourses around “crisis” are socially constructed and often take on a life of their own (Widmaier et al. 2007, p. 755). Social constructivists criticize the materialist treatment of crisis events, for even exogenous shocks must be interpreted endogenously. While crisis often involves a state of emergency, Merkel (2020) also identifies forms of “latent crisis,” for example, when describing the gradual erosion of democracy and the deterioration of democratic institutions. Similar features pertaining to the permanent nature of crises have been identified in the realm of international theory. According to Debrix (2008), crises represent a permanent constitutive feature of social relations. Nabers (2019, p. 269) comes to a similar conclusion by drawing on Zygmunt Baumann and Carlo Bordoni, who both argue that we live in times of constant crisis.

Poststructuralists such as Nabers (2017, p. 419) perceive crises less as actor dependent and more as an “underlying principle of society” where “the transformation of society rests on a continuous encounter with crisis.” Crises must then be perceived as the dislocation of formerly stable hegemonic discourses. A new signifier, such as terrorism, destabilizes the internal structure of the existing discourse. In American discourse, this comprises notions such as freedom or stability. Such forms of dislocation are inherent in societies and part of the production of subjectivity and radically contingent nature of crisis (Nabers 2019, p. 272).

To summarize, while materialist approaches view crisis as a set of exogenous events which can be analyzed in objective ways, social constructivists, pragmatists, and critical theorists treat crisis as “human constructions of ambiguous contingencies through which meaning is imposed” (Spector 2019, p. 277). From a history of meaning perspective, “crisis” has been used to describe an exceptional situation, which includes something akin to a turning point, but also an “immanent, permanent condition of the world,” e.g., when describing the history of Christian salvation (Koselleck and Richter 2006, p. 398).

## Dimensions of crisis in arms control

In the early days of IR, the materialist, analytical approach of crisis dominated. International crises were either framed as changes in decision-making within a nation or, at the system level, as behavioral changes in the interaction patterns between states (Hermann 1972). For Young (1967: 10), an international crisis is “a set of rapidly unfolding events which raises the impact of destabilizing forces in the general system or any of its subsystems substantially above ‘normal’ (i.e., average) levels and increases the likelihood of violence occurring in the system.” McCormick (1978, p. 356) proposes combining the two and perceives international crisis as a situation that is defined by the perceived condition of a threat, surprise, and short decision time, as well as by behavioral change in the interaction patterns between nations. International crisis was perceived as a situation of high risk, placing states or even the international system of states in danger of entering into conflict and war. The most visible and possibly most dangerous international crisis was the Cuban Missile Crisis in 1962. Crisis and arms control seem to be inextricably related. That said, crisis in arms control can have many different facets and does not often culminate in an international crisis.

Arms control and disarmament aim at reducing the dangers of conflict escalation and war (Müller and Schörnig 2006). Disarmament serves to reduce the number of weapons and ultimately bans entire weapons categories, e.g., chemical weapons or antipersonnel mines. Arms control can also contribute to disarmament. For the most part, however, it seeks to stabilize international relations through equal limits or reductions, confidence building, transparency, and verification. Arms control during the Cold War primarily aimed to reduce tensions and prevent nuclear war between the two superpowers. Arms control crises frequently emerged during the Cold War as well. The United States, for example, refused to ratify SALT II after the Soviet invasion of Afghanistan and the Soviet Union broke off INF talks after NATO allies deployed new intermediate-range systems to Western Europe. The Conference on Disarmament experienced stalemates on a variety of arms control issues during that time. In U.S. domestic politics, arms control has frequently become a matter of partisan controversy—the success or failure of arms control was often decided by party politics (Böller 2021; Jacobson 1984). In retrospect, changes in decision-making in the United States and the Soviet Union as well as behavioral changes in the relations between the two superpowers affected the bilateral arms control process. Moreover, the “security imaginaries” of the two superpowers contributed to arms control crises, since exogenous events were endogenously interpreted (Weldes 1999). This example already illustrates how the materialist and the socially constructed perspectives of arms control crises are often intertwined.

Today, the bilateral nuclear architecture is in a profound and protracted crisis (Sauer 2020). In 2001, the United States withdrew from the Anti-Ballistic Missile Treaty and in 2019 from the INF Treaty. In a renewed period of great power rivalry, the United States, Russia, and China are all pursuing ambitious armament programs and modernizing their nuclear arsenals. The deterioration of great power relations also affects multilateral arms control and disarmament fora, such as the First Committee of the UN General Assembly where debates on the status of the different arms control regimes take place each fall. The power rivalries between China, Russia, and the United States dominated the last meeting in 2020 (Acheson 2020) and continue to negatively affect disarmament and nonproliferation regimes, such as the NPT, the Biological Weapons Convention, and the Chemical Weapons Convention. Another indicator of the deterioration of interstate relations is the increase in military budgets. In 2021, global military expenditure rose to an unbelievable two trillion U.S. dollars (SIPRI 2022).

Since 2012, the Assad government’s repeated use of chemical weapons in the Syrian war has posed a significant challenge to the Chemical Weapons Convention. While diplomats saw the regime as being in crisis, academic research points to the considerable normative stability of the chemical weapons taboo and the CWC (Jakob 2019; Price 2019; Rosert 2019).

Crisis can also occur in more hidden, latent forms as gradual erosion takes place. Fifty years after entering into force, the NPT is perceived as going through a “midlife crisis” (Kimball 2020) and also appears to be suffering from a functional crisis as well as a crisis of legitimacy (Dembinski/Peters 2020). Many non-nuclear weapon states have become increasingly dissatisfied with the failure of the five nuclear weapon states to fulfill their NPT Article VI obligations. Calls for the NPT to be ditched in favor of the new TPNW (Pretorius and Sauer 2021) could worsen the crisis. Over time, core components of the conventional arms control regime in Europe were also eroded. The CFE Treaty is dysfunctional since Russia suspended its participation in 2007. Not long after this, the 2008 war in Georgia and the Russia’s 2014 annexation of Crimea led to a further deterioration of the European cooperative arms control architecture (Finaud 2020; Zellner 2020). With the U.S. withdrawing from Open Skies, the entire regime is on the verge of collapse. As these multiple and often parallel crises demonstrate, political linkages—e.g., between bilateral nuclear arms control and conventional arms control in Europe—can serve as additional catalysts, creating unwanted bridges between different domains of arms control. These linkages further complicate the future of arms control (Kühn 2020).

As mentioned above, crisis can also become a policy-enabling motive for establishing new arms control agreements. This understanding connects to an earlier understanding of crisis in 1920s Germany when it became synonymous with the potential for positive change (Graf 2020, p. 25). Such an understanding of crisis can also be applied to different arms control agreements. The dissatisfaction with the NPT was a huge motive for the conclusion of the TPNW in 2017. The “crisis motive” has also been an important driving force in the negotiations on humanitarian arms control (Wisotzki 2013). The human suffering caused by uncontrolled and indiscriminate use and dispersion of certain conventional weapons has been the main motive used by networks of transnational non-governmental organizations to lobby for and persuade states to conclude the 1997 Mine Ban Treaty, the 2001 UN Program of Action on the Illicit Trade of Small Arms and Light Weapons (UNPoA), the 2008 Cluster Munition Convention, as well as the ATT in 2013. Networks of transnational non-governmental organizations constructed their campaigns around the crisis motive by drawing attention to the human suffering caused by these weapons.

This brief overview of the different dimensions of crisis in arms control already provides an insight into the connections between material facts and social constructivist dimensions of crisis. The term “crisis” has been used to describe exceptional situations, but also to refer to the more latent and gradual erosion of arms control regimes. Many of the Cold War regimes have gradually slipped into functional crises as well as crises of legitimacy. While the use of the word crisis often signals a situation with a negative outcome, the depiction of crisis can also generate agency and facilitate a new beginning. Such an optimistic understanding of crisis has experienced a serious set-back due to the Russian war in Ukraine (See Chap. 5).

## Overview of the special issue

In this Special Issue, we concentrate on crisis in arms control by drawing on diverse empirical examples from across the different fields. While the previous section provided an overview of the multiple dimensions of crisis in arms control, the individual articles of this Special Issue delve much deeper into the various arms control regimes and seek to decipher the different layers of crisis.

The Special Issue opens with Jana Baldus, Harald Müller, and Carmen Wunderlich exploring the different layers of crisis in multilateral nuclear arms control, disarmament, and nonproliferation. The absence of meaningful disarmament under the NPT has led to the latter’s steady erosion—regime conflicts are a visible sign of this, but the emergence of the TPNW also challenges the entrenched power relations between nuclear weapon states and non-nuclear weapon states. Moreover, cases of nuclear proliferation in East Asia and the Middle East have added to compliance and enforcement crises. In order to stabilize the nuclear nonproliferation regime and find ways out of the crisis, the authors recommend cooperative solutions to the current great power struggle, overcoming the differences between the factions within the NPT, and finding ways of strengthening the core principle behind nuclear arms control: to prevent nuclear war.

Ulrich Kühn focusses on the crisis of nuclear arms control between the United States and Russia. Recalling past successes and failures of that process during the Cold War, he identifies three key factors that influenced this distinctive bilateralism: a shared willingness to shield the bilateral process from political disruption, U.S. bipartisan support, and the ability to cooperatively address the vertical diffusion of offensive and defensive missile capabilities. During the twenty years of crisis, from 2001 to 2021, these key factors underwent significant changes and ultimately had a negative effect on the bilateral process, which, he concludes, makes this crisis unique compared to earlier episodes of regression. Most importantly, both sides were no longer willing to shield the bilateral process in its entirety during this period, including defensive and sub-strategic offensive elements. With a view to future consequences, his findings point to reduced arms race stability, weaker negotiated outcomes, and an erosion of the global nonproliferation regime.

The great breakthrough in conventional arms control in Europe took place when the Cold War was almost over and marked the beginning of another protracted crisis. Alexander Graef argues that today’s crisis of conventional arms control in Europe runs deeper. He identifies three dimensions of this existential crisis. Beyond the long-term erosion of the conventional arms control architecture in Europe he points to intensified cleavages in political relations between the cooperating parties. These two dimensions of crisis are further boosted by technological weapons innovations, such as unmanned aerial systems, sophisticated jamming technology, and long-range precision strike capabilities.

The crisis of European arms export control also shows some continuities. Simone Wisotzki and Max Mutschler argue that, in many ways, the crisis is a permanent one, where collective efforts to regulate a policy area and implement standards and rules that have been agreed on consistently fail. The ineffectiveness of the EU Common Position on arms exports was particularly evident during the two review processes where member states failed to agree on meaningful revisions. The crisis of effectiveness has also led to a crisis of legitimacy. Non-governmental and civil society organizations organize protests and successfully file court cases against arms export practices violating international humanitarian law and human rights law. Moreover, the Europeanization of arms production places additional pressure on the system, and EU member states continue to have different policy practices when it comes to national arms export licensing.

The regulation of emerging technologies, such as lethal autonomous weapons systems, remains a difficult undertaking. In their article, Christian Alwardt and Niklas Schörnig argue for forms of preventive arms control to “keep the genie in the bottle” and propose looking into existing arms control knowledge. A crisis situation might ultimately occur if such automated technologies are used without any meaningful human control. However, NGOs and states differ when it comes to possible approaches to arms control. While NGOs call for a ban, many states deem such a strict form of regulation unrealistic. Alwardt and Schörnig argue that states should think back to the security benefits of arms control during the Cold War and the inherent concepts of such a regime in order to develop effective arms control solutions rather than lobbying for a ban. Classical concepts of arms control, such as verifiable compliance, will have to be adapted to meet the new challenges stemming from emerging technologies.

The “crisis motive” advanced many agreements in the area of humanitarian arms control. This was also the case when states agreed to the UNPoA in 2001. Non-governmental organizations and many like-minded states demonstrated that the lack of control and the vast illicit proliferation of small arms and light weapons (SALW) had led to a protracted humanitarian crisis affecting states and populations alike. Twenty years later, however, the UNPoA is still a long way from solving the crisis, Simone Wisotzki argues. While the UNPoA’s standards and rules are broad, significant unregulated loopholes remain, such as ammunition, civilian possession, and non-state actors. Verification provisions are also weak, and states are less and less willing to fulfill their basic reporting obligations. Global solutions, such as the UNPoA, suffer from serious problems, such as the discrepancy between outcome documents of conferences and practical implementation measures. However, regional and subregional approaches seem to be more tailored to the specific and distinct needs of states and civil society in order to tackle SALW proliferation.

## Russia’s aggression against Ukraine

A final word of caution must be added to this introduction: As stated at the beginning, all the articles in this Special Issue were written and completed before Russia invaded Ukraine on February 24, 2022. This fact makes this Special Issue at once timely and untimely. It makes it untimely because none of the authors were able to explore the implications that this unprovoked aggression might have for international arms control and disarmament efforts. Yet, it makes this Special Issue timely because German peace and conflict researchers, in particular, had been warning us for years that the crisis of arms control is not just another sign of deteriorating great power relations but contributes directly to diminished global security and stability (Kühn 2020; Pieper 2020; Wisotzki 2020; Müller and Tokhi 2019).

Now that the Russian leadership has broken with the post-Cold War European peace and security order, rebuilding a new order containing supporting elements of arms control will be extremely difficult. For years to come, NATO and Russia could face a military standoff along a new dividing line from the far north of Finland to the Turkish Bosporus Strait. Whether that standoff will remain “cold” or turn into a “hot” war is too early to predict. What is predictable is that, from a global perspective, Russia’s violation of Ukrainian sovereignty and territorial integrity just adds another significant element of stress to the already strained nuclear nonproliferation regime—if only due to the very uncomfortable fact that Ukraine had returned Soviet nuclear arms to Russia in exchange for Russian security guarantees, including territorial integrity. Other states might recalibrate their security strategy outlook and reflect on possible proliferation. Whether the bilateral Russian-American arms control dialog will survive this pivotal moment is far from certain right now. At the same time, all this makes arms control, risk reduction, and confidence- and security-building measures politically all the more pressing.

Future research on arms control will have to relearn some historical lessons, e.g., from the Cold War, and start thinking anew about stabilizing precarious military relations in the context of conventional and nuclear deterrence and reassurance. At the same time, advocates of disarmament and nonproliferation—of weapons of mass destruction and conventional arms alike—will need to grapple with militaristic narratives aimed at denouncing such efforts as untimely or downright naïve. However, a future plagued, yet again, by conventional and nuclear arms races would consume the time and energy urgently needed to collectively tackle climate change or prevent future pandemics. Realistically, the global community does not actually have the time or resources for another large-scale military standoff with Russia and perhaps even with China. The task for future research on arms control is therefore to contribute to conceptual ideas on how to overcome the crisis and to free political and intellectual resources for more pressing global problems. We hope that the following articles will still contribute to this vital debate.

## Footnotes

1 António Guterres, Remarks Before the Conference on Disarmament, 25.02.2019, www.un.org/sg/en/content/sg/speeches/2019-02-25/remarks-the-conference-disarmament.
